# Trends in Kaposi's sarcoma-associated Herpesvirus antibodies prior to the development of HIV-associated Kaposi's sarcoma: A nested case-control study

**DOI:** 10.1002/ijc.29329

**Published:** 2014-11-14

**Authors:** Katie Wakeham, W Thomas Johnston, Angela Nalwoga, Emily L Webb, Billy N Mayanja, Wendell Miley, Alison M Elliott, Denise Whitby, Robert Newton

**Affiliations:** 1 Medical Research Council/Uganda Virus Research Institute, Uganda Research Unit on AIDSEntebbe, Uganda; 2 Epidemiology and Cancer Statistics Group, Department of Health Sciences, University of YorkHeslington, York, United Kingdom; 3 Institute of Cancer Research, University of GlasgowScotland, United Kingdom; 4 Department of Infectious Disease Epidemiology (ELW), London School of Hygiene and Tropical MedicineBloomsbury, London, United Kingdom; 5 Department of Clinical Research (AME), London School of Hygiene and Tropical MedicineBloomsbury, London, United Kingdom; 6 Viral Oncology Section, AIDS and Cancer Virus Program, Leidos Biomedical, Frederick National Laboratory for Cancer ResearchFrederick, MD; 7 International Agency for Research on CancerLyon, France

**Keywords:** HIV-associated Kaposi's sarcoma, sub-Saharan Africa, Kaposi's sarcoma associated-herpesvirus, HIV, AIDS

## Abstract

HIV-associated Kaposi's sarcoma (KS) is a public health challenge in sub-Saharan Africa since both the causative agent, Kaposi's sarcoma associated-herpesvirus (KSHV), and the major risk factor, HIV, are prevalent. In a nested case-control study within a long-standing clinical cohort in rural Uganda, we used stored sera to examine the evolution of antibody titres against the KSHV antigens K8.1 and latency-associated nuclear antigen (LANA) among 30 HIV-infected subjects who subsequently developed HIV-related KS (cases) and among 108 matched HIV/KSHV coinfected controls who did not develop KS. Throughout the 6 years prior to diagnosis, antibody titres to K8.1 and LANA were significantly higher among cases than controls (*p* < 0.0001), and titres increased prior to diagnosis in the cases. K8.1 titres differed more between KS cases and controls, compared to LANA titres. These differences in titre between cases and controls suggest a role for lytic viral replication in the pathogenesis of HIV-related KS in this setting.

Kaposi's sarcoma (KS) is currently the most commonly reported cancer in Uganda and in other parts of sub-Saharan Africa^1^ where both the underlying cause, Kaposi's sarcoma associated-herpesvirus (KSHV), and a key risk factor, HIV, are prevalent. Well-established assays to diagnose KSHV infection rely on the detection of antibody responses to KSHV-specific antigenic proteins, which are preferentially expressed during viral latency or lytic replication. The major protein markers of KSHV latency and lytic replication are encoded by latency-associated nuclear antigen (LANA) and K8.1, respectively.^2^ In case-control studies from South Africa and Uganda, high antibody titres against KSHV have been observed in patients with KS compared to controls.^3^,^4^ Longitudinal studies in North America and Northern Europe report that elevated antibody titres against KSHV antigens LANA and/or K8.1 are present prior to the onset of clinically evident KS.^5^–^8^ However, there are no longitudinal studies describing the evolution of antibodies against KSHV prior to the development of KS from the African continent, despite both virus and tumor being relatively frequent there. The epidemiology of KSHV and KS differ between sub-Saharan Africa and the US and Europe. In Uganda for example, KSHV infection can be acquired during childhood,^9^ many years prior to HIV infection, and estimates of the seroprevalence of KSHV range between 36% and 60%.^9^–^13^ In contrast, in the US and Northern Europe, the estimates of KSHV prevalence are in general lower (<10%),^14^,^15^ and infection with both KSHV and HIV often occurs during adult life.^16^ These differences may impact on the development of antibody responses to KSHV and on the pathogenesis of KS.

Among individuals with HIV-associated KS in sub-Saharan Africa survival is generally poor.^17^,^18^ The limited prognosis of these individuals can be explained in part by lack of access to early diagnosis of both HIV and KS and in part by limited treatment options. The introduction of antiretroviral therapy (ART) in the US and Europe has been associated with a substantial decrease in both the incidence and severity of newly diagnosed KS in HIV-infected patients.^19^,^20^ However, access to ART remains limited in Uganda; in 2011, ART coverage for people with advanced HIV infection was about 50%.^21^ Therefore, especially in resource limited settings, identifying those at greatest risk of HIV-associated KS may be useful for the development of both therapeutic and preventative strategies to address this important public health concern.

**What's New?**

Infection with Kaposi sarcoma associated-herpesvirus (KSHV) and HIV, the major risk factor for Kaposi sarcoma, is common in sub-Saharan Africa. However, little is known about the evolution of KSHV antibody responses in HIV-infected individuals prior to the clinical onset of KS. This study shows that antibody titres against KSHV antigens K8.1 and latency-associated nuclear antigen (LANA) increase significantly in the six years leading up to HIV-associated KS. Titres of K8.1, a lytic antigen, rose more than LANA titres, indicating that the activation of genes in the lytic cycle of viral replication is important in KS development.

To further our understanding of the patterns of antibody responses to KSHV over time, we conducted a case-control study nested within an actively followed population-based clinical cohort of HIV-infected adults living in rural Uganda.^22^–^24^ We investigated whether the pattern of KSHV antibody titres over time in individuals who subsequently develop KS differs from the KSHV antibody titre pattern among those who do not develop KS.

## Material and Methods

### Study population

Our study was nested within an actively followed longitudinal clinical cohort of HIV-infected adults. These individuals were identified from within a larger general population cohort (GPC), established in 1989 in rural South West Uganda, to study the population dynamics of HIV disease in a typical rural Ugandan community.^25^,^26^

HIV-infected adults identified within the GPC were invited to join the Rural Clinical Cohort (RCC)^22^–^24^ at diagnosis and were reviewed in the study clinic at three-monthly intervals. A clinical questionnaire was completed at each visit and a full physical examination carried out and documented. ART, available from 2004, was commenced in accordance with Ugandan Ministry of Health criteria^27^: CD4 count of 200 cells per μL or below; or WHO clinical stage 4; or advanced WHO clinical stage 3 with persistent or recurrent oral thrush and invasive bacterial infections; or CD4 count of 250 cells per μL or below during pregnancy. The first-line ART regime was zidovudine (AZT), lamivudine (3TC) and nevirapine (NVP). CD4 cell counts were measured (FACSCount, Becton Dickinson, San Jose, CA) at baseline, and at every clinic visit a serum sample was collected and stored at −80°C at Medical Research Council/Uganda Virus Research Institute (MRC/UVRI) Clinical Diagnostic Laboratory in Entebbe, Uganda. Patients on ART had an HIV viral load measurement (Amplicor MONITOR 1.5, Roche Molecular Systems, NJ) at drug commencement and approximately every 6 months thereafter.

We identified, through the RCC study database, 30 individuals diagnosed with HIV-associated KS, between 2 October 1990 and 5 July 2010. All cases were confirmed by a review of clinic notes conducted independently by two clinicians. Five cases had histological verification of diagnosis, and the remainder had clinical documentation only. Controls were selected from among HIV-seropositive adults without a diagnosis of KS during the study period using the following procedure: each case was classified by sex, age band (≤ median age of 37 years or ≥38 years) and CD4 count band (≤200 cells per μL, 201–500 cells per μL, ≥501 cells per μL) at the time of their KS diagnosis. Potential controls were then examined to find the time points at which their characteristics (sex, age band and CD4 count band) matched those of the case, and this time point was then taken as the pseudodiagnosis date for that control, which for simplicity will be referred to as the KS diagnosis date hereafter. Next, to ensure that all controls were KSHV seropositive at KS diagnosis, those fulfilling matching criteria were tested for KSHV antibodies using the methods described below, and 149 were found to be KSHV seropositive to either K8.1 or LANA at KS pseudodiagnosis. For each case, up to four individually matched controls were selected from the pool of 149 HIV and KSHV seropositive people, and they were included in the dataset as matched to the corresponding case. Results for HIV viral load were considered a suitable representation of an individual's viral load at KS diagnosis if measured within 100 days.

### Laboratory procedures

All available serum samples taken up to and including the KS diagnosis sample were tested for antibodies against KSHV using a quantitative lytic K8.1 ELISA and latent LANA ELISA as previously described.^2^,^28^ Serum samples positive for antibodies against K8.1 at a 1:20 dilution were subsequently tested at doubling dilutions from 1:20 to 1:20,480 to measure titre. Serum samples positive for antibodies to LANA at a 1:100 dilution were tested at doubling dilutions from 1:100 to 1:102,400. The titre value was the dilution before which the sample lost positive signal. For analysis, the titre values were replaced by a digit representing the number of dilutions, from zero for a KSHV seronegative sample up to 10 for K8.1 and up to 11 for LANA. Individuals blinded to patient details performed the KSHV ELISA assays at the UVRI and at the Viral Oncology Section, Frederick National Laboratory for Cancer Research.

### Statistical analysis

The characteristics of cases and of controls were initially compared using a Mann–Whitney test, *t*-test or *χ*^2^ test as appropriate. The change in titres (defined as the number of doubling dilutions) prior to KS diagnosis were analyzed using linear regression adjusted for matching factors age, sex and CD4 count. Correlation between repeated measurements on individual patients was adjusted for using generalized estimating equations (GEE) with an exchangeable correlation structure. To graphically present trends in titre, the GEE model was used to predict mean titres for cases and controls from a population of HIV-positive individuals in a 1:1 sex ratio, with a median age of 37 years and a CD4 count of between 200 and 500 cells per ml at KS diagnosis. Variability between individual patients was explored by constructing a multilevel mixed effects regression model incorporating random elements for both the titre level, the trend over time and covariance between the two. Random effects from the mixed models for each individual were estimated using the best linear unbiased predictor. Interactions between time prior to KS diagnosis and case-control status were examined in both models to assess whether patterns in titre over time differed between cases and controls. Tests for statistical significance were derived from likelihood ratio test statistics. All *p* values were two-sided, and significance was considered at the 5% level. Analyses were carried out using Stata 12SE (StataCorp LP, College Station, TX).

### Ethical approval

Ethical approval for our study was obtained from the UVRI Science and Ethics Committee and from the Uganda National Council for Science and Technology.

## Results

**Figure 1:**
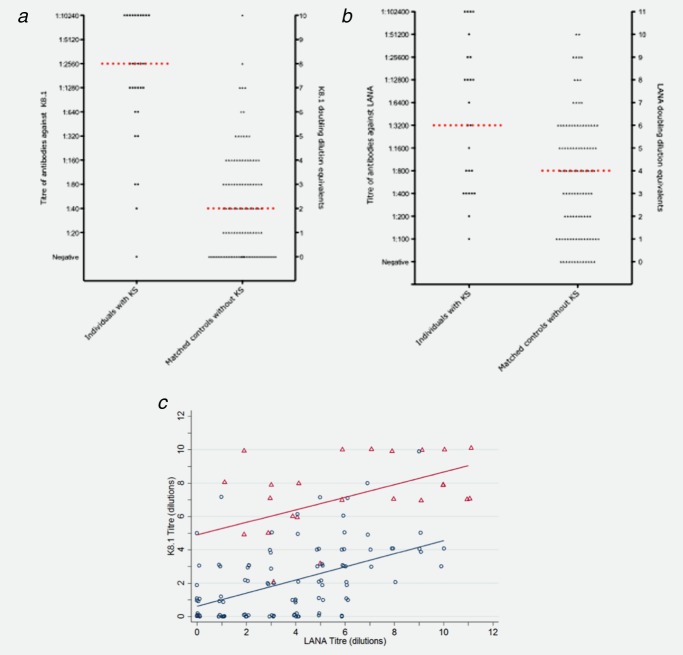
Distribution of titres of antibodies to K8.1 (A) and LANA (8), and doubling dilution equivalents, is 30 HIV-positive individuals with Kaposi's sarcoma (KS) and 108 matched controls, at time of diagnosis (for cases) of pseudo-diagnosis (for controls). For each patient with KS, up to four controls without KS were selected matched for sex, CD4 count group and age. The median titre in each group is indicated by a horizontal red line. Scatter plot of K8.1 versus LANA titre (C) with linear fits plotted separately for the cases and controls.

Results were available for 30 cases and 108 matched controls. The median number of samples available from each individual was 3 (range 1–12). All cases at diagnosis and controls at pseudodiagnosis were seropositive to either K8.1 or to LANA but not necessarily to both. At the time of KS diagnosis 93% (28/30) of the cases were seropositive to K8.1 compared to 69% (74/108) of the controls (*p* = 0.006). Titres to K8.1 were higher among KS cases than controls at KS diagnosis (2,640 [interquartile range (IQR) 660–10,240] vs. 40 [IQR 0–80], *p* < 0.0001, Fig. 1, Table 1). Similar, but less marked differences in seroprevalence (*p* = 0.045) and titre (3,200 [IQR 200–102,400] vs. 800 [IQR 0–25,600], *p* < 0.0001) were observed for the LANA antigen at the KS diagnosis time point: 100% (30/30) of cases were seropositive compared to 88% (95/108) of the controls (Fig. 1, Table 1). There was significant positive correlation between K8.1 and LANA titres at KS diagnosis among both cases (*p* = 0.50, *p* < 0.02) and controls (*p* = 0.48, *p* < 0.0001, Fig. 1*c*).

**Table 1:** Characteristics of KS patients at diagnosis and control patients at pseudo-diagnosis

	Case	Control	
Factor	*n* = 30	*n* = 108	*p* value
Sex, *n* (%)			
Female	14 (47%)	52 (48%)	Matching factor
Male	16 (53%)	56 (52%)	
Age, *n* (%)			
Less that 37 years	14 (47%)	56 (52%)	Matching factor
Older than 37 years	16 (53%)	52 (48%)	
CD4 count class, *n* (%)			
Less than 200	16 (53%)	60 (56%)	
200–500	8 (27%)	24(22%)	Matching factor
Greater than 500	6 (20%)	24 (22%)	
Median CD4 count at diagnosis, (IQR)	131 (40–468)	177 (109–491)	0.09
On ART at time of diagnosis, *n* (%)	7 (23%)	21 (19%)	0.6^1^
Comparison of K8.1 results between cases and controls			
Median K8.1 titre at diagnosis (IQR)	2640 (660–10,240)	40 (0–80)	<0.0001
Median K8.1 titre of all samples (IQR)	320 (80–2,560)	40 (0–80)	<0.0001
Comparison of LANA results between cases and controls			
Median LANA titre at diagnosis (IQR)	3200 (200–102400)	800 (0–25,600)	<0.0001
Median LANA titre of all samples (IQR)	3200 (100–102,400)	800 (0–12,800)	<0.0001

*p* value calculated using Ranksum test, unless otherwise stated

1 *χ*^2^ test

**Figure 2:**
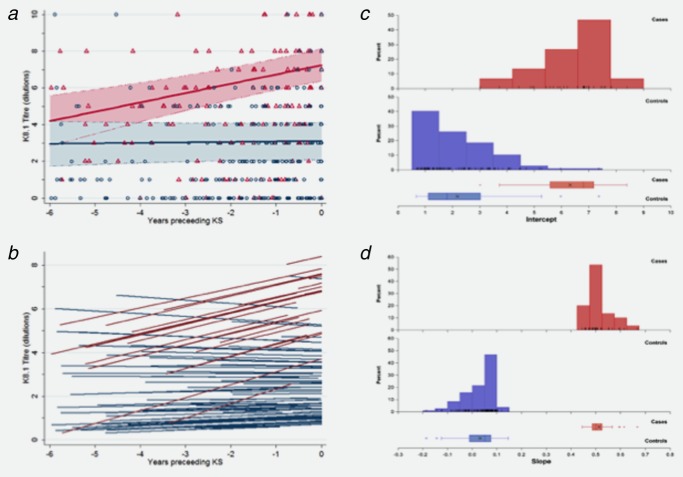
K8.1 titres in the six years prior to diagnosis (for cases) or pseudo-diagnosis (for controls). (A) for Kaposi's sarcoma (KS) cases (red symbols and lines) and non-KS controls (blue symbols and lines) with predictions bases on the GEE (A) and random effects (B) regression models presented in Table 2. GEE regression model predictions are based on a group of patients in a 1:1 sex ratio, at the median of 37 years at diagnosis and with a C04 count between 200 and 500 cells/ml; the shaded areas represent the 95% confidence intervals. Random effects model predictions are the fitted lines for each individual patient with at least 2 measurements. Distributions of individual intercept (C) and slope (D) estimates from the random effect model. Red bars/boxes denote KS cases and blue bars/boxes denote control patients.

There was a significant interaction for K8.1 antibody titres between case-control status and time, such that the difference in K8.1 titres between cases and controls was largest at the time points closest to KS diagnosis (Table 2). Estimates from GEE and random effects models were similar for all parameters (Table 2). Citing the GEE model results: at the time of diagnosis, K8.1 titres were approximately 16-fold higher among patients developing KS (4.1 doubling dilutions, 95% CI: 3.39–4.88) than controls. There was no significant time trend in titres among the control patients (0.02 doubling dilutions per year, 95% CI: −0.10–0.15); however, titres among KS patients increased approximately twofold every 2 years (0.48 doubling dilutions per year, 95% CI: 0.23–0.73) (Table 2, Fig. 2*a*).

**Table 2:** Comparing K8.1 and LANA titres of HIV patients who have developed KS to those who have not developed KS

		K8.1						LANA					
		GEE		Random effects^1^			GEE			Random effects^1^			
Factors		Estimate (95% CI)		*p*	Estimate (95% CI)		*p*	Estimate (95% CI)		*p*	Estimate (95% CI)		*p*
KS patient *vs*. Control^2^^a^		4.13	(3.39,4.88)	<0.001	4.14	(3.34,4.94)	<0.001	2.29	(1.10,3.48)	<0.001	2.2	(1.16,3.25)	<0.001
Years prior to diagnosis^2^^b^		0.02	(−0.10,0.15)	0.69	0.03	(−0.11,0.17)	0.66	0.08	(−0.13,0.29)	0.48	0.006	(−0.13,0.15)	0.93
Case-Time interaction^3^		0.48	(0.23,0.73)	<0.001	0.48	(0.24,0.73)	<0.001						
Sex (Female *vs*. Male)		−0.6	(−1.26,0.51)	0.07	−0.53	(−1.17,0.11)	0.1	0.07	(−0.79,0.93)	0.87	−0.08	(−0.96,0.79)	0.85
Age at diagnosis		0.02	(9.75x10^−5^,0.04)	0.05	0.02	(−0.002,0.04)	0.08	0.02	(−0.009,0.05	0.18	0.02	(−0.008,0.06)	0.14
CD4 count	200–500 cells per ml	0.73	(−0.19,1.64	0.12	0.71	(−0.07,1.48)	0.07	0.21	(−0.97,1.39)	0.72	0.26	(−0.81,1.33)	0.63
(vs <=200 cells/ml)	>500 cells per ml	−0.71	(−1.43,0.02)	0.06	−0.68	(−1.54,0.17)	0.12	0.2	(−0.85,1.25)	0.71	0.19	(−0.95,1.33)	0.74
Random effects^4^	Intercept				2.02	(1.30,3.16)					6.99	(5.26,9.29)	
	Slope				0.005	(0.001,0.16)					0.29	(0.19,0.45)	
	Covariance				−0.1	(−0.27,0.06)					0.7	(0.27,1.14)	

Serum samples of cases prior to KS diagnosis are compared to samples of control prior to pseudo-diagnosis using linear regression

1 covariance between the two estimated random effects (base titre and time effect per patient) was found to contribute only to the LANA model but has been retained in the K8.1 model.

2 due to the significant interaction in the models of K8.1 these factors may be interpreted as (a) the difference in titre between case and control patients at (pseudo)diagnosis (*i.e*. time=0) and (b) the time trend among patients who did not develop KS.

3 in the K8.1 models this factor is interpreted as the difference in change in titre between case and control patients over time; not significant in LANA models and therefore not included.

4 variance estimates

From the random effects model, predicted individual K8.1 antibody dynamics were clearly different for KS cases and controls (Fig. 2*b*). Intercept estimates ranged from 3.0 to 8.4 dilutions among people with KS and were lower among controls (range 0.7–7.4 dilutions), with similar variance estimates within the two groups (Fig. 2*c*). While there was substantial overlap in distributions the shapes of the distributions were different. The slope estimates had no overlap between KS cases and controls: case K8.1 titres increased at rates between 0.44 and 0.67 dilutions per year whereas rates of change among controls ranged from a decrease of −0.19 dilutions per year to an increase of 0.14 dilutions per year (Fig. 2*d*). Thus, groups were easily distinguished based on slope. Variability in slope estimates among cases was similar to the variance among controls. There was a small negative covariance for K8.1 between the intercept (antibody titre at diagnosis) and slope (rate of change in antibody level) but this did not contribute significantly to the model fit.

**Figure 3:**
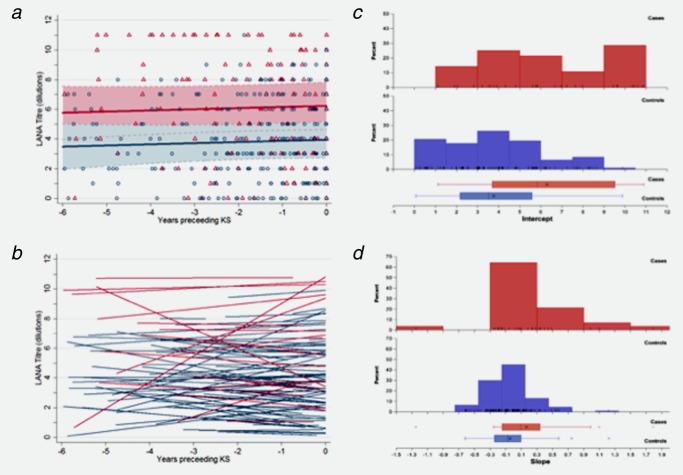
LANA titres in the six years prior to diagnosis (for cases) or pseudo-diagnosis (for controls). (A) for Kaposi's sarcoma (KS) cases (red symbols and lines) and non-KS controls (blue symbols and lines) with predictions bases on the GEE (A) and random effects (B) regression models presented in Table 2. GEE regression model predictions are based on a group of patients in a 1:1 sex ratio, at the median of 37 years at diagnosis and with a C04 count between 200 and 500 cells/ml; the shaded areas represented the 95% confidence intervals. Random effects model predictions are the fitted lines for each individual patient with at least 2 measurements. Distributions of individual intercept (C) and slope (D) estimates from the random effect model. Red bars/boxes denote KS cases and blue/boxes denote control patients.

The two LANA models were also comparable. From the GEE model, patients with KS had LANA titres approximately five times higher (2.29 doubling dilutions; 95% CI: 1.10–3.48) than patients who did not have KS; however, there was no trend in the titres over time and there was no significant interaction between the effects of KS status and time (Table 2, Fig. 3*a*). Predicted individual LANA titre dynamics of cases and controls were comparable (Fig. 3*b*). Distributions of patient intercepts and slopes overlapped substantially; intercepts ranged from 1.00 to 10.81 dilutions among KS cases and from 0.11 to 9.92 dilutions among controls (Fig. 3*c*); rates of change in LANA titre (slope) ranged between −1.29 and 1.77 dilutions per year among individuals with KS and −0.60 and 1.26 dilutions per year among controls (Fig. 3*d*). There was a positive covariance for LANA between intercepts and slopes indicating that individuals with higher titres at diagnosis had higher rates of change of antibody level over time (Fig. 3*b*).

Given the infrequent use of ART (Table 1) and consequently the few viral load measurements available, adjusting for ART status and HIV viral load at time of KS diagnosis did not materially change the results of any of the models (data not shown).

## Discussion

This is the first longitudinal study examining patterns of antibody titres to both K8.1 and LANA in individuals with HIV-associated KS, living in a KSHV-endemic population in sub-Saharan Africa. The study has shown that individuals who develop HIV-associated KS have higher K8.1 and LANA antibody titres compared to those who do not, and that this is evident up to 6 years prior to KS diagnosis. In addition, K8.1 antibody titre increased with time toward KS diagnosis among cases, whereas individuals who did not develop KS had relatively stable titres over time.

The results presented here are in accord with studies conducted among cohorts based in the West; antibody titres against KSHV antigens are higher among cases and increase prior to tumor diagnosis.^5^,^6^,^8^,^16^,^29^ Prospective studies measuring both lytic and LANA KSHV antibodies by ELISA are lacking. However, one study of HIV-infected people in the United Kingdom reported an increased risk of KS with increasing LANA antibodies measured by ELISA.^6^

Like all herpesviruses, KSHV cycles between two distinct phases: latency, a quiescent state of persistent infection essential for the development of KS, and lytic reactivation, characterized by ordered increased gene transcription and in most cases, viral replication and host cell death.^30^ In KS lesions, most infected cells are latently infected and express only the latency-associated genes including the antigen encoded by LANA.^31^ However, a small population of cells (<5%) also express lytic antigens including K8.1, indicating that lytic gene expression has a role in KS carcinogenesis.^32^ Many of the genes implicated in oncogenesis such as vGPCR are expressed during the lytic cycle. Clinical studies have also suggested the potential importance of lytic gene activation in KS development. In a clinical trial treatment with oral or intravenous ganciclovir, a drug that blocks lytic but not latent KSHV infection reduced the incidence of KS.^33^–^35^

The raised K8.1 titres among individuals who develop HIV-associated KS found in our study is in accord with the evidence that lytic gene expression may be important in KS tumorigenesis. It is likely that cellular immune responses are also critical to KS risk, as has been shown for transplant-associated KS.^36^ Future studies examining the interplay between KSHV viral load, antibody titre and cellular immune responses are warranted.

Our study had a number of limitations. The definition of case-control status in our study relied, in the majority of cases, on clinical diagnoses made at the time of a regular clinic visit. While large dermal KS lesions are relatively easy to diagnose, lack of histological confirmation may have resulted in some misclassification of cases and controls and small dermal lesions may have been missed. Further, no diagnostic tests were available to ensure that controls did not have visceral KS involvement or another KSHV-related pathology. Date of KS diagnosis was based on the clinical review date, which may have been affected by how regularly the HIV clinic was attended. Only simple linear titre dynamics could be investigated in our study because the majority of patients had only three samples available. The results of our study also rely on the validity of the measured antibody titre. The relatively small numbers of individuals on ART at KS diagnosis, in spite of low CD4 counts, reflects local policy (treatment of individuals with CD4 T-cell counts below 200 per µl) and limited access to ART in Uganda at the time when these cases were diagnosed.

In our study, K8.1 titres provided a more useful means of differentiating patients developing KS from those who did not, compared to LANA titres. The differences in titre between cases and controls were sufficiently pronounced that the findings might have some clinical utility in terms of predicting which individuals may develop HIV-associated KS. K8.1 titres showed more marked differences than LANA titres and the slope of change in K8.1 titres completely distinguished cases from controls, suggesting that this may be a particularly useful parameter for such a purpose. In further research, development of a simple assay and investigation as to whether these parameters could be of use in other patient groups (such as transplant recipients) might be considered.

For the related gammaherpesvirus, Epstein-Barr virus, age at infection is known to be an important determinant of outcome.^37^ The fact that KSHV titres are higher among cases, compared to controls, so long prior to diagnosis, might suggest that events early in KSHV infection determine subsequent risk of disease; age at infection with KSHV may be related to this but whether this is a key determinant of KSHV load is not known. Our results suggest that investigation of this, and identification of risk factors associated with elevated KSHV load, is relevant to understanding KS risk and pathogenesis.

In conclusion, during the 6 years prior to KS diagnosis, antibody titres against KSHV antigens K8.1 and LANA were significantly higher among cases compared to controls and K8.1 titres increased as diagnosis approached. These findings suggest strategies for identifying and understanding KS disease risk and support the hypothesis that lytic gene expression and replication are important in the pathogenesis of HIV-related KS in this setting.
